# SCN10A Mutation in a Patient with Erythromelalgia Enhances C-Fiber Activity Dependent Slowing

**DOI:** 10.1371/journal.pone.0161789

**Published:** 2016-09-06

**Authors:** Andreas M. Kist, Dagrun Sagafos, Anthony M. Rush, Cristian Neacsu, Esther Eberhardt, Roland Schmidt, Lars Kristian Lunden, Kristin Ørstavik, Luisa Kaluza, Jannis Meents, Zhiping Zhang, Thomas Hedley Carr, Hugh Salter, David Malinowsky, Patrik Wollberg, Johannes Krupp, Inge Petter Kleggetveit, Martin Schmelz, Ellen Jørum, Angelika Lampert, Barbara Namer

**Affiliations:** 1 Institute of Physiology and Pathophysiology, Friedrich-Alexander-Universität Erlangen-Nürnberg, Erlangen, Germany; 2 Section of Clinical Neurophysiology, Department of Neurology, Oslo University Hospital -Rikshospitalet, Oslo, Norway; 3 AstraZeneca R&D, Södertälje, Sweden; 4 Department of Anesthesiology, Friedrich-Alexander-Universität Erlangen-Nuremberg, Erlangen, Germany; 5 Department of Neuroscience, Clinical Neurophysiology, Uppsala University, Uppsala, Sweden; 6 Institute of Physiology, RWTH Aachen University Hospital, Aachen, Germany; 7 AstraZeneca R&D, Cambridge, United Kingdom; 8 Department of Anesthesiology Mannheim, Heidelberg University, Mannheim, Germany; Universitatsklinikum Wurzburg, GERMANY

## Abstract

Gain-of-function mutations in the tetrodotoxin (TTX) sensitive voltage-gated sodium channel (Nav) Nav1.7 have been identified as a key mechanism underlying chronic pain in inherited erythromelalgia. Mutations in TTX resistant channels, such as Nav1.8 or Nav1.9, were recently connected with inherited chronic pain syndromes. Here, we investigated the effects of the p.M650K mutation in Nav1.8 in a 53 year old patient with erythromelalgia by microneurography and patch-clamp techniques. Recordings of the patient’s peripheral nerve fibers showed increased activity dependent slowing (ADS) in CMi and less spontaneous firing compared to a control group of erythromelalgia patients without Nav mutations. To evaluate the impact of the p.M650K mutation on neuronal firing and channel gating, we performed current and voltage-clamp recordings on transfected sensory neurons (DRGs) and neuroblastoma cells. The p.M650K mutation shifted steady-state fast inactivation of Nav1.8 to more hyperpolarized potentials and did not significantly alter any other tested gating behaviors. The AP half-width was significantly broader and the stimulated action potential firing rate was reduced for M650K transfected DRGs compared to WT. We discuss the potential link between enhanced steady state fast inactivation, broader action potential width and the potential physiological consequences.

## Introduction

Inherited erythromelalgia (IEM) is a chronic pain syndrome which is linked to mutations in voltage-gated sodium channels (Navs), mostly in the subtype Nav1.7 [1,2]. Patients suffer from recurring attacks of burning pain in their extremities, reddening and warming of the skin. Typical triggers are mild exercise, elevated ambient temperature or sometimes simply wearing of clothes. The pain can often only be relieved by cooling. In contrast to the inherited form, secondary erythromelalgia (EM) often manifests following other diseases, such as polycythemia or diabetes and typically has a later onset in the patient’s life.

Quantitative sensory testing (QST, [3], [4]) of EM or IEM patients in pain free intervals does not always show hyperalgesia, even if these patients carry mutations that were shown to cause pronounced hyperexcitability in DRG cells [5]. Often, comparatively few abnormalities are described during the relatively pain free intervals and these abnormalities are often compatible with a small fiber neuropathy (SFN), such as elevated thermal thresholds indicating hypofunction or fiber degeneration [6–8]. In a previous microneurography study analyzing patients with EM, spontaneous activity in C-nociceptors and mechanically sensitized C-fibers were described [5,9]. In recordings from patients with polyneuropathy, more spontaneous activity of the so-called”silent” C-nociceptors (CMi-nociceptors) were observed in those with pain compared to those without pain [10]. In microneurography, activity dependent slowing (ADS) of action potential conduction is most likely caused by increased inactivation of Navs [11], although intracellular sodium ion accumulation may also occur and has been linked to reduced excitability of axons in a recent modelling study [12]. Thus, ADS may be used as an indirect readout for reduced availability and excitability of Navs in single C-fibers of awake human patients. Indeed, in one EM patient with the I848T mutation in Nav1.7, activity-dependent *speeding* of conduction was observed [5]: increased stimulation frequency resulted in activity dependent speeding, i.e. conduction velocity increased [13,14]. This indicates the existence of a positive feedback between nociceptor activity and hyper-excitability instead of the regular ADS (activity dependent *slowing*).

Of the nine known Nav subtypes several are expressed in DRGs: the TTX sensitive (TTXs) Nav1.1, Nav1.2, Nav1.6 and Nav1.7, and the TTX resistant (TTXr) variants Nav1.9 and Nav1.8, the latter of which is predominantly expressed in nociceptive neurons [15–18]. TTXs channels are thought to amplify subthreshold depolarizations and action potential (AP) initiation [19], whereas Nav1.8 is thought to be responsible for high frequency firing [20].

To date, more than 20 mutations in *SCN9A*, the gene encoding for Nav1.7, have been linked to IEM in several affected families [1,21]. Almost all of them induce a hyperpolarizing shift of the voltage-dependence of activation, rendering the expressing cells more susceptible to small depolarizing stimuli. In two cases, instead of activation, steady-state fast inactivation is shifted to more depolarized potentials leading to increased excitability of the expressing cells [22,23]. Transfection of dissociated DRGs with IEM mutations revealed that mutant expressing neurons respond to supra-threshold stimulation with a higher firing rate than WT expressing cells (e.g.: [24–28]). Recently, rare variants of genes other than SCN9a were described in patients with clinically verified erythromelalgia: those genes included SCN10A (Nav1.8), SCN11A (Nav1.9), SCN5A (Nav1.5), SCN7A (Nav2.1), SCN8A (Nav1.6), SCN1B (Nav β1 subunit), SCN3B (Nav β3 subunit), TRPA1, TRPV1, WNK1 and NGFR [8].

Here we provide a detailed description of the p.M650K mutation of the TTXr Nav1.8 [8] through its clinical phenotype, microneurography findings, voltage-clamp and current-clamp analysis, offering insights into the role of Nav1.8 in ADS and the generation of peripheral pain in an erythromelalgia patient.

## Methods

### Clinical assessment, neurological and genetic testing

The patient was referred to the Section of Clinical Neurophysiology, Oslo University Hospital for neurophysiological evaluation of distal pain in her extremities. The patient underwent neurological and neurophysiological examinations, including QST (thermal thresholds), EMG/neurography and microneurography. The diagnosis of EM was based on the presence of episodic pain, warm and red feet and pain alleviated by cold, the absence of indicators of small fiber neuropathy in thermotesting and the absence of other causes for small fiber neuropathy like diabetes mellitus, polycythemia vera or medication (see Results for details, and [6]). A thorough medical history was inquired with focus on previous and family history of pain and details about actual pain symptoms, including start and evolution of pain, characteristics and intensity on a numeric scale from 0 to 10, occurrence of spontaneous episodic and evoked pain, eliciting and alleviating factors. The clinical and neurological examination consisted of an inspection of the extremities and of an evaluation of sensory function (light touch with cotton swab and a von Frey hair testing, pain sensation with a needle and vibration with a Rydel Seiffer tuning fork), and motor function (muscle strength and assessment of tendon reflexes from the upper and lower extremities).

Genetic testing was performed as previously described [8] and showed a heterozygous single-point mutation in SCN10A which was not found in 96 controls (it is also listed as very rare in the ExAC browser: http://exac.broadinstitute.org/variant/3-38783939-A-T). The study was approved by the regional ethical committee REK (Regional Committees for Medical and Health and Health ethics) South East, record-number S-07204a. The individual in this manuscript has given written informed consent (as outlined in PLOS consent form) to publish these case details.

### Quantitative sensory testing

Threshold temperatures for the sensation of warmth, cold, heat pain and cold pain were determined using a computerized Thermotest (SENSElab, Somedic A/B, Hörby, Sweden) with a thermode size of 5 x 2.5 cm. Warmth detection threshold (WD), cold detection threshold (CD), heat pain detection threshold (HP) and cold pain detection threshold (CP) were determined from a baseline temperature of 32°C with a 1°C/s rate of change. The patient was instructed to push a signal-button when the relevant sensation was perceived. When this happened or if cut-off temperature (50 C and 10 C) was obtained, the temperature returned to baseline. WD, CD, HP, CP were determined from the thenar eminence, the dorsum of both feet and both legs at knee-level. For calculations of thresholds, an average of five recordings for WD and CD and the average of three recordings for HP and CP were used. The values were compared with normal values from the neurophysiological department of Rikshospitalet University Oslo.

### EMG/neurography

A Keypoint EMG apparatus (Denmark) was employed, measuring motor amplitude, distal latency and conduction velocity of the median, ulnar, peroneal and posterior tibial nerves, and sensory amplitude and conduction velocity of the median, ulnar and sural nerves. EMG of proximal and distal muscles form the right leg was performed, assessing the properties of the motor unit potential as searching for possible signs of denervation. The values were compared to normal Keypoint data.

### Microneurography recording technique

**Fig 1:**
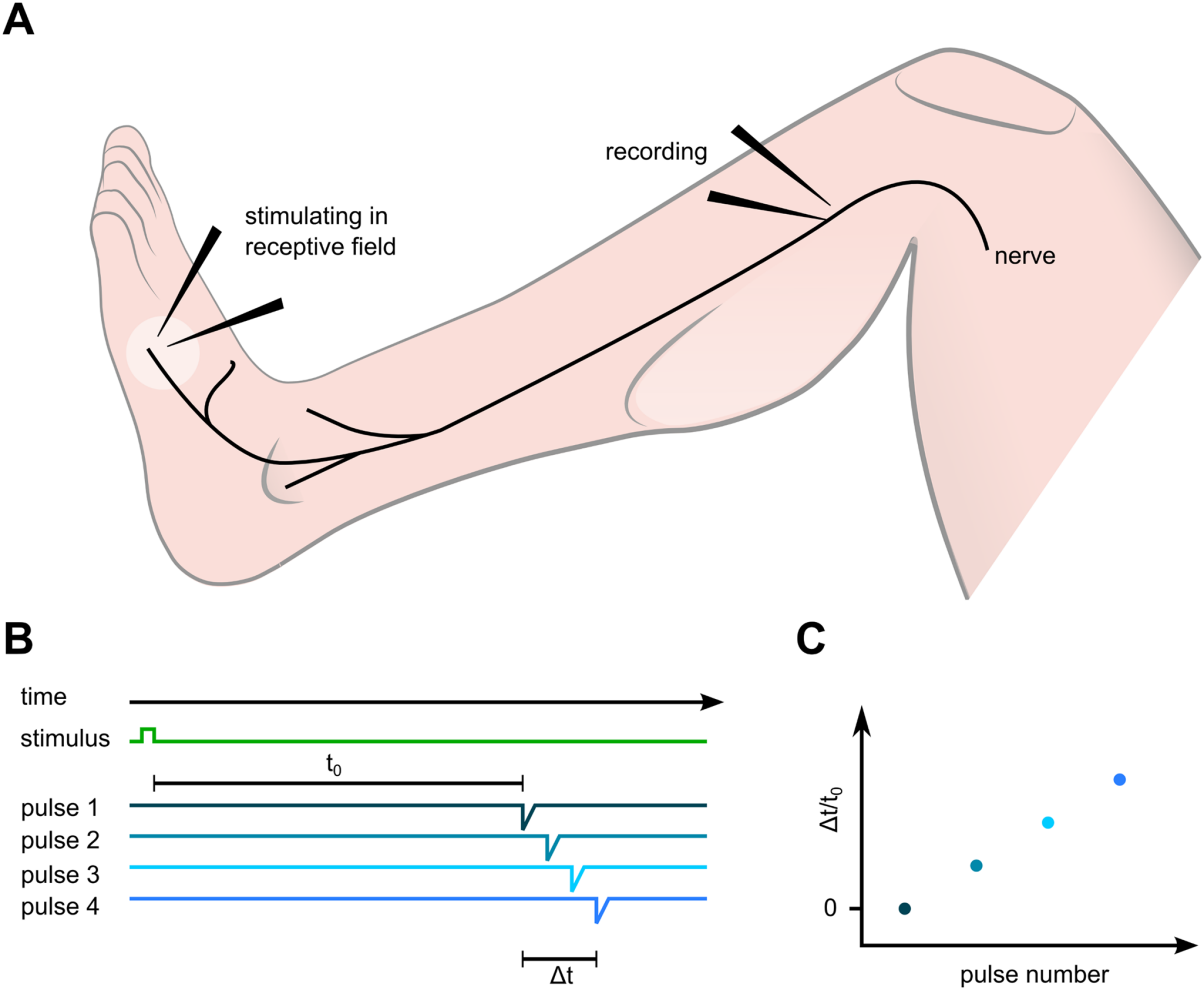
A: Microneurography setup. Action potentials are evoked electrically in the receptive field of the pierced fascicle, travel proximally along the peroneal nerve and are recorded at the fibular head. B: The interval between the first electrical stimulus and the arrival time of the evoked AP at the recording needle t_0_ is set as reference conduction latency. Conduction latencies of subsequent pulses (Δt) are calculated relative to the first latency (Δt/t_0_). C: Plot of the relative conduction latency (Δt/t_0_) versus pulse number. The increase in Δt/t_0_ indicates the slowing of the AP conduction upon repetitive stimulation.

APs from single C-fibers were recorded by microneurography from the peroneal nerve at the level of the fibular head. Recordings from five EM patients without Nav mutations were used as controls and the data were compared to microneurography data from healthy controls which were published before (Namer et al 2009). The principle of microneurography has been described in detail previously [29] and is shown in Fig 1. Briefly, a tungsten electrode (Frederick Haer, Bowdoinham, ME, USA) was inserted into a fascicle of the peroneal nerve containing cutaneous C-fibers close to the knee. Individual C-fibers were identified by applying electrical pulses transdermally in the innervation area of the nerve on the foot dorsum, evoking APs with low conduction velocities (<2 m/s, Fig 1A). Two stimulation needles (0.15 mm diameter) were then inserted in the skin receptive field of the C-fibers of interest. The C-fibers were stimulated repetitively (0.25 Hz, 0.5 ms, 1–30 mA) via a constant current stimulator (DS7A; Digitimer, Hertfordshire, England, UK) through the stimulation needles and the resulting APs were recorded, amplified, processed online, and stored to a computer using custom-written Spike2 software and a micro1401 DAC (CED, Cambridge, UK, Fig 1B). The data were analyzed off-line by Drever software (Uppsala, Sweden).

### Response to natural stimulation and spontaneous activity

When a C-fiber was activated repetitively at low frequencies through stimulation needles in the receptive field of the recorded fibers, APs with stable conduction latencies were recorded. However, additional activity in this C-fiber, e.g. due to mechanical stimulation, induced a sudden increase in conduction latency, a phenomenon known as marking [29]. Such increase in latency is due to an activity-dependent slowing of conduction velocity (ADS) in C-fibers [30] and shown in Fig 1B and 1C. Marking was used to assess the responses in single C-fibers to natural (mechanical, heat) and electrical stimulation. Spontaneous activity of a fiber was judged by an abrupt increase of response latency followed by a gradual normalization (marking) without external stimulation or evidence of a unidirectional block.

### Electrical stimulation protocols

After a 2 min pause in the electrical stimulation, activity dependent changes in conduction velocity were induced either by applying a low-frequency stimulation protocol (20 pulses at 0.125 Hz, 20 pulses at 0.25 Hz, and 30 pulses at 0.5 Hz) through the stimulation needles in the skin or a high-frequency protocol consisting of a train of 360 electrical pulses at 2 Hz. ADS was measured at the 3^rd^, 10^th^, 120^th^ and 360^th^ (last) pulse of the high frequency protocol. Total ADS was calculated as the relative increase in latency from the first pulse to the last in both protocols. By switching to the lower stimulation frequency of 0.25 Hz the total ADS gradually returns to baseline (“recovery phase”). During this phase we measured the response latency for the 10^th^ pulse at 0.25 Hz and normalized it to the total ADS (rate of recovery). The value was excluded if spontaneous activity interfered with ADS or rate of recovery.

### Natural stimulation protocols

Fibers were tested for mechanical response by stimulating with a 75 g von Frey filament in the receptive fields. Fibers which responded to this stimulation by marking were classified as mechano-positive.

Heat response was assessed by using a feedback controlled bulb delivering a heat ramp to the skin, starting at 32°C with an increase of 0.25°C/sec up to a maximum of 50°C. Fibers responding by marking were classified as heat positive. Heat threshold was determined if possible.

### Classification of C-fibers

The classification of single C-fibers was based on ADS and the response to natural stimulation. C-nociceptors were classified as CM-nociceptors if they were mechano-positive (von Frey 75 g) and had less than 3% total ADS in the low-frequency protocol, or as CMi-nociceptors if they were mechano-insensitive (von Frey 75 g) and had more than 5% total ADS in the low frequency protocol. The remaining C-nociceptors were classified as C-nociceptors of unknown type (not possible to classify as normal CM or CMi).

C-fibers were defined as sympathetic C-fibers if they had characteristic reversal of the initial ADS [31] in the high-frequency protocol and/or were activated by sympathetic stimulation.

### Cell culture and transfection

Wistar rat pups (3–6 days of both sexes, bred at FAU Erlangen-Nuremberg, Germany) were anaesthetized with halothane (Sigma-Aldrich GmbH, Steinheim, Germany) and decapitated in accordance with ethical guidelines established by German animal protection law, approved by the animal protection committee of Regierung Mittelfranken, Germany, DIVA-number 095621001683, registered according to regulation (EG) number 1069/2009, DE09562000821. After decapitation, the backbone was removed rapidly and bisected longitudinally in ice-cold PBS. DRGs were removed, desheathed, cleaned and enzymatically dissociated in Dulbecco’s modified Eagle's Medium (DMEM, Gibco-Life technologies, New York, USA) supplemented with collagenase/protease for 45 min at 37°C. Cells were mechanically triturated with small diameter Pasteur pipettes in TNB medium (Biochrom AG, Berlin, Germany) and transfected using the nucleofector II (Lonza group Limited, Basel, Switzerland) according to the manufacturers protocol. Poly-D-Lysine (Sigma-Aldrich GmbH, Munich, Germany) coated cover slips were loaded with the cell suspension and after cells settled for 5–10 min TNB medium was added. DRGs were incubated at 37°C and 5% CO_2_ for one day before experiments were performed.

ND7/23 neuroblastoma cells (kindly provided by Stephen G. Waxman) were maintained in DMEM medium (Gibco-Life technologies) containing 4.5 g/l Glucose, 10% fetal bovine serum (Biochrom AG) and 1% Penicillin/Streptomycin (PAA Laboratories GmbH). One day before transfection ND7/23 cells were plated on 3.5 cm dishes and transfected with Nanofectin (PAA Laboratories GmbH) according to the manufacturers protocol using 1 μg hNav1.8 in pIRESpuro3 vector and 0.5 μg EGFP-C1 (Clontech Laboratories, Inc., Mountain View, USA). Cells were recorded 20 to 30 h after transfection.

### Whole-cell patch-clamp recordings

Whole-cell voltage and current clamp recordings were performed using glass electrodes with tip resistances of 1.5 to 2.2 MΩ, manufactured with a DMZ puller (Zeitz Instruments GmbH, Martinsried, Germany) and filled with an internal solution comprising for current-clamp recordings on DRGs (in mM): 135 K-Gluconate, 4 NaCl, 10 KCl, 10 HEPES, 2 Na_2_ATP, 0.4 Na_3_GTP (adjusted to pH 7.3 with KOH), and for voltage-clamp experiments on ND7/23 cells (in mM): 140 CsF, 10 NaCl, 10 HEPES, and 1 EGTA (adjusted to pH 7.38 with CsOH). Activation properties of Nav1.8 expressed in ND7/23 cells were determined using a pipette solution with low NaCl to shift the reversal potential to more depolarized voltages (in mM): 140 CsF, 2 NaCl, 10 HEPES, 10 TEA-Cl and 1 EGTA (adjusted to pH 7.38 with CsOH).

The external bathing solution contained for DRGs (in mM): 140 NaCl, 4 KCl, 1 MgCl_2_, 2 CaCl_2_, 10 HEPES, 5 Glucose (adjusted to pH 7.4 with NaOH), for ND7/23 cells (in mM): 140 NaCl, 1 MgCl_2_, 1 CaCl_2_, 10 HEPES, 10 Glucose, 10 TEA-Cl (pH 7.4, adjusted with NaOH). In experiments with ND7/23 cells, endogenous TTXs Navs were suppressed by adding 1 μM TTX (Biotrend, Wangen/Zurich, Switzerland) to the bath solution.

Only small (< 25 μm) DRGs were chosen for recordings. Size was measured as the maximal diameter of the cells on an image taken with a digital camera at the patch clamp setup. Recordings were performed at room temperature (22 ± 1°C). Current-clamp recordings were sampled at 20 kHz (low-pass filter 6 kHz) using an Multiclamp 700B amplifier in conjunction with a Digidata 1322A interface and pClamp10 software (all from Molecular Devices, Sunnyvale, USA). A HEKA EPC-10 USB operated with Patchmaster software (HEKA electronics, Lambrecht, Germany) was used for voltage-clamp signal recording, low-pass (< 10 kHz) filtering and digitizing (100 kHz).

Pipette potential was zeroed prior to seal formation and capacitive transients were compensated using C-fast for pipette-capacity correction and subsequently C-slow for cell-capacity compensation, the series resistance was compensated by at least 50%. The absolute peak current (pA) was divided by the capacitance as determined by C-slow compensation to obtain current densities. For voltage-clamp recordings, leak pulses were applied following the test pulse, and mean leak current was subtracted digitally online corresponding to the P/4 leak pulse procedure.

Voltage protocols were carried out after current stabilization. Standard current-voltage (I-V) curves were recorded using 100-ms pulses from a holding potential of −120 mV to a range of potentials (-100 to +80 mV) in 10 mV steps. Conductance-voltage curves were obtained by calculating the conductance (G) at each voltage (V) using the equation G = I/(V − V_rev_), with V_rev_ being the reversal potential, determined for each cell individually. Conductance-voltage curves were fitted with a Boltzmann equation: G_Na_ = G_Na,max_/(1 + exp [(V_m_ − V_half_)/k]), where G_Na_ is the voltage-dependent sodium conductance, G_Na,max_ is the maximal sodium conductance, V_half_ is the potential at which activation is half-maximal, V_m_ is the membrane potential, and k is the slope factor. Steady-state fast inactivation was determined by applying a 500 ms pulse with various potentials (ranging from -130 mV to +10 mV in 10 mV steps) and determining the remaining available current using a depolarizing pulse to 0 mV. Currents were normalized to the maximum inward current. Inactivation decay time constants were determined by single-exponential fits to the current decay of the current obtained with the activation protocol. We provide all single, processed recordings shown in the data figures in S1 and S2 Files.

### Statistics

An unpaired two-sample Student’s t-test was used to test for statistical significance for the patch-clamp data. Data are presented as mean ± standard error of the mean (SEM) unless otherwise specified.

Microneurography data were processed using Statistica version 26. Due to the exploratory design and the small size and distribution of the data obtained from microneurography, descriptive data (median and 25th and 75th percentiles) are presented and significance testing was performed using the non-parametric Mann-Whitney-U-test.

## Results

### A patient carrying the Nav1.8 M650K mutation shows clinical signs of erythromelalgia

We describe here a 53 year old woman with symptoms beginning at the age of 35 for whom genetic testing revealed the heterozygous amino acid substitution M650K, Methionine (M) to Lysine (K), in her SCN10A gene encoding for Nav1.8 [8]. No additional mutation was identified in her other sodium channel or candidate genes. Her father and sister report burning feet, but were not diagnosed with EM, nor genetically tested. The patient has previously suffered from migraine-like attacks and has had neck problems. Her present symptoms are described as episodes of painful warm feet several times a week, predominantly in the evenings and early night. The feet are red and feel warm, but the skin temperature appears normal upon clinical examination. Her symptoms are worse after physical activity and after exposure to heat (e.g. warm floors). Her pain is superficial and of a burning and pressing character, only located in the feet, predominantly the soles. Pain intensity was rated up to 5 on a numeric rating scale from 0 to 10 where 0 is no pain and 10 maximal pain. The patient does not receive medication but her pain is relieved by cooling. Therefore she often walks barefooted or submerges the feet and legs in cold water. She had normal findings by EMG/neurography and QST (thermal thresholds) (see Tables 1 and 2) and also no abnormalities in her clinical and neurological findings, except for a reduced sensibility for light touch (von Frey test) on the feet from the toes to just above the ankle. As we observed normal nerve conduction results for the sural nerves we excluded a large fiber sensory neuropathy.

**Table 1:** Results of QST (thermal thresholds) of the IEM patient carrying the Nav1.8/M650K mutation.

	WD	CD	HP	CP
*Right thenar*	33.4	30.6	42.6	11.3
*Right dorsum foot*	36.8	27.8	44.9	11.4
*Left dorsum foot*	37.1	29.6	44.3	11.9
*Right knee level*	39.6	29.2	47.3	11.4
*Left knee level*	36.1	30.3	44.3	11.2

WD: warm detection threshold, CD: cold detection threshold, HP: pain threshold, CP: cold pain threshold. All values are given in °C.

**Table 2:** Sensory nerve conduction properties of the sural nerve of the IEM patient carrying the Nav1.8/M650K mutation.

*Nerve- Motor fibers*	Latency (ms)	Amplitude (mV)	CV (m/s)
*Median right*	2.7 (-1.8)	6.9 (-0.7)	58.8 (0.0)
*Ulnar right*	2.2 (-2.3)	9.7 (-0.1)	63.2 (0.3)
*Tibial right*	3.5 (-1.4)	7.4 (-0.9)	56.0
*Peroneal right*	3.1 (-1.9)	4.9 (-0.5)	55.0
*Tibial left*	3.0 (-2.0)	15.8 (1.2)	58.7
*Peroneal left*	3.4 (-1.5)	4.9 (-0.5)	52.3
***Nerve- Sensory fibers***			
*Median right*	2.6	13 (0.2)	64.3 (1.0)
*Ulnar right*	2.1	12 (0.3)	63.0 (0.6)
*Sural right*	2.2	7.8 (-0.8)	59.9 (1.1)
*Sural left*	2.6	9.9 (-0.4)	60.0 (1.1)

CV: conduction velocity. Mean values ± SD are shown.

### Patient’s nociceptors show low rate of spontaneous activity

We examined a total of 25 C-fibers of this patient by microneurography, including 20 C-nociceptors (ten CM-nociceptors, eight CMi-nociceptors, two C-nociceptors of unknown type, Table 3), two sympathetic C-fibers and three fibers that could not be identified. In order to assess the specific impact of the Nav1.8 M650K mutation, we chose to compare our findings in this patient with previously published EM patients without identified Nav mutations (23 CM-nociceptors, eight CMi-nociceptors, 14 C-nociceptors of unknown type and 11 sympathetic C-fibers). For direct comparison with a healthy control group we included data of previously published healthy age matched controls [32].

**Table 3:** Basic sensory and axonal properties of CM and CMi-fibers.

	M650K		EM no mutation	
	CM	CMi	CM	CMi
*Spont. Act.*	1 (10)	1 (8)	5 (23)	4 (8)
*Mechanic response*	10 (10)	2 (8)	23 (23)	0 (8)
*Heat response*	10 (10)	2 (8)	16 (17)	5 (5)
*Heat threshold (°C)*	44.00 (3) (42.00–44.00)	-	42.50 (13) (37.75–44.25)	43.00 (3) (42.00–43.00)
*CV (m/s)*	1.05 (0.97–1.08)	0.70 (0.59–0.83)	1.00 (0.95–1.04)	0.61 (0.51–0.73)
*ADS_1/8_* (%)	0.24 (0.16–0.27)	1.27 (1.07–1.95)	0.21 (-0.02–0.36)	1.30 (1.06–1.59)
*ADS_tot_* (%)	1.48 (0.82–2.22)	6.64 (5.99–9.17)	1.74 (1.02–2.32)	6.68 (6.22–7.10)
*Rec10 (%)*	42.42 (31.45–55.95)	18.50 (15.20–20.88)	40.23 (30.86–55.04)	12.95 (11.16–17.01)

ADS was induced by a 0.125 Hz stimulation for 20 pulses, followed by a 0.250 Hz stimulation for 20 pulses followed by 0.5 Hz stimulation for 30 pulses. The total ADS (ADS_tot_) is the amount of normalized ADS at the last pulse of this protocol. Rec10 is the 10^th^ pulse after this protocol during 0.25 Hz stimulation. ADS_1/8_ is the amount of ADS at the 20th electrical stimulation pulse of the 0.125 Hz stimulation period. Total numbers of tested fibers are stated in parentheses, and in addition the 25^th^ and 75^th^ percentiles, where appropriate.

Two nociceptors with biophysical properties typical for CMi-nociceptors, showed a response to mechanical stimuli. These nociceptors were interpreted as mechanical sensitized CMi-nociceptors. In contrast to the mechanical sensitization in CMi-nociceptors, the mechanical responses of six of the ten CM nociceptors were reduced in comparison to mechanical responses of healthy subjects: CM-nociceptors could only be activated by the first stimulus, and the response itself was small and not of a typical bursting character.

Spontaneous activity was observed in one CM- and one CMi-nociceptor (10.0% of total C-nociceptors) of the M650K patient. Due to the indirect assessment of spontaneous activity in microneurography via the marking method it is not possible to give exact measures about the frequencies of spontaneous discharges, but from the pattern of markings spontaneous activity appeared to be of lower frequency when compared to markings obtained during mechanically or heat induced bursting. In recordings from the EM control group, 42.2% of the C-nociceptors fired spontaneously (5 CM, 4 CMi, 10 C-nociceptors of unknown type). Thus, the patient carrying the M650K mutation displayed fewer spontaneously active fibers compared to EM patients without Nav mutation.

### Patient’s CMi nociceptors display enhanced ADS

Conduction velocity and ADS at the low-frequency protocol in CM- and CMi-nociceptors were comparable to EM-controls (see Table 3). Conduction velocity and ADS of the two sympathetic C-fibers of the M650K patient were normal (Table 2) and in the range of the sympathetic fibers of the EM-controls.

**Fig 2:**
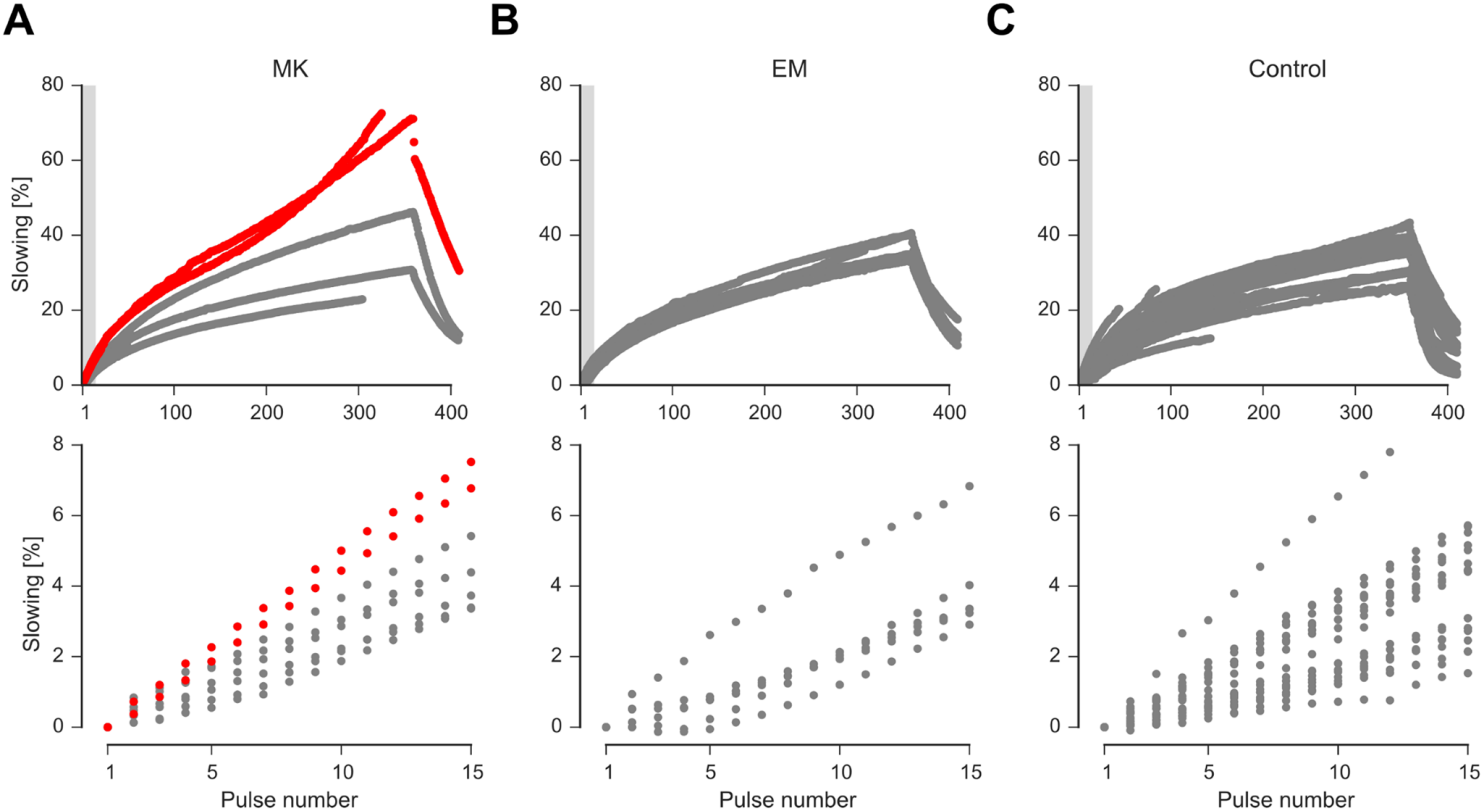
ADS during 2 Hz stimulation protocol of CMi-nociceptors in the M650K patient (A) and EM controls without Nav mutations (B) and age-matched healthy controls (C). The lower panel shows the first 15 pulses of the upper panel (subset indicated with gray shadow in the upper panel). Note the prominent abnormal progressive increase in ADS in the last part of the stimulation in two of the CMi-nociceptors from the M650K patient (red), none in EM patients without mutation and only one less pronounced in age-matched healthy controls showed this behavior.

However, in contrast to what was displayed during the low frequency protocol, the CMi-nociceptors of the patient carrying the M650K mutation displayed more ADS during 2 Hz stimulation compared to the EM-controls and healthy subjects, even during the first pulses of the protocol (Table 4 and Fig 2A–2C, lower panel). Two CMi-fibers of the M650K patient showed an abnormal increase in ADS at the end of the high-frequency protocol (Fig 2A). There were no noticeable differences between the CM-fibers of the three groups (Table 4).

**Table 4:** ADS and recovery of the C-nociceptors during high-frequency protocol.

Patient	M650K		EM no mutation		Healthy	
Nociceptor class	CM	CMi	CM	CMi	CM	CMi
3rd pulse	0.15 (0.13–0.19) (n = 9)	0.76 (0.32–1.09) (n = 8)	0.14 (0.10–0.17) (n = 18)	0.58 (0.03–1.21) (n = 6)	0.16 (0.14–0.23) (n = 23)	0.45 (0.23–0.76) (n = 19)
10th pulse	0.90 (0.60–1.27) (n = 9)	2.95 (2.17–4.25) (n = 8)	0.93 (0.74–1.36) (n = 18)	2.09 (1.97–4.20) (n = 6)	1.12 (0.69–1.54) (n = 23)	2.15 (1.46–3.36) (n = 19)
120th pulse	13.56 (12.14–14.74) (n = 6)	25.47 (16.90–31.03) (n = 5)	15.78 (12.62–17.33) (n = 16), *	20.08 (18.53–21.63) (n = 5), *	15.71 (14.51–17.89) (n = 23), *	21.97 (19.09–23.06) (n = 19)
360th pulse	21.63 (19.04–24.40) (n = 5)	46.20 (30.27–71.06) (n = 3)	23.86 (21.76–26.54) (n = 14)	34.88 (33.45–39.90) (n = 5)	27.11 (24.98–30.64) (n = 20), *	37.35 (31.77–39.34) (n = 17)

Note, that the ADS values of the M650K patient are overall higher than in the groups of EM patients without mutation and a healthy age-matched control. The 25th and 75th percentiles and the total number of tested fibers are shown in parentheses,

* p<0.05,

U-test.

Thus, ADS in the patient with the M650K mutation is enhanced compared to controls and to patients with EM without Nav mutation already during the first pulses of the 2 Hz stimulation and leads to an abnormal progressive increase in ADS towards the end of the 2 Hz stimulation in two CMi-nociceptors.

### Nav1.8 M650K mutation reduces sensory neuron firing rate

**Fig 3:**
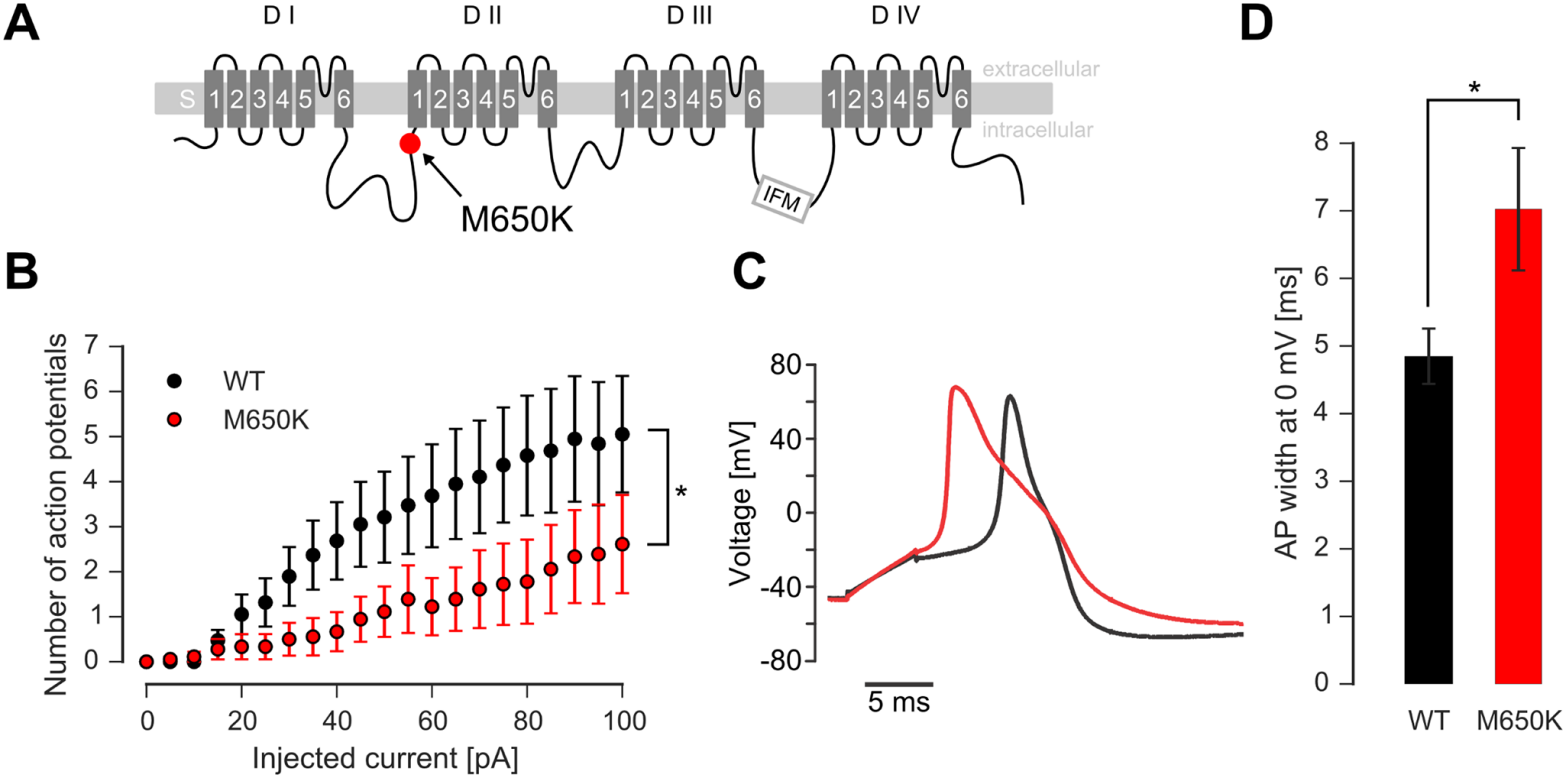
The M650K mutation decreases the firing rate of DRG neurons. A: Schematic overview of a eukaryotic Nav showing the four domains DI to DIV, each consisting of the transmembrane segments S1 to S6. The rare genetic variant M650K in Nav1.8 (red dot, [8]) is located at the adjacent intracellular side of DII/S1. B: Number of APs evoked by 500 ms square step current injections to WT (black markers) or M650K (red markers) transfected cells (n = 18 and 19). * < 0.05 in a 2-sided t-test C: Example of an AP recorded from a WT (black trace) and a M650K (red trace) transfected DRG neuron evoked by a 5 ms depolarizing pulse. Injected current was twice threshold current. D: The AP half width was significantly broader in M650K than in WT transfected neurons (n = 13 and 19). *< 0.05 in a 2-sided t-test.

The mutation M650K which was found in our patient is located at the intracellular side of DIIS1 in the linker between DI and DII of Nav1.8 (Fig 3A). In order to assess the impact of the mutation on cellular excitability, we transiently transfected dissociated rat pup DRGs with Nav1.8 or its M650K mutation. Surprisingly, neurons transfected with the M650K mutation produced significantly fewer APs compared to WT when stimulated with a 500 ms square current pulse (Fig 3B). Single step evoked APs revealed that transfection of the M650K mutation broadened the AP half width at 0 mV from 4.9 ± 0.4 ms to 7.0 ± 0.9 ms (Fig 3C and 3D, p<0.05 in a two sided t-test). Resting membrane potential remained unaffected (-41.0 ± 0.8 mV for WT versus -43.2 ± 1.6 mV for the M650K mutation, not significantly different). Thus, the Nav1.8 M650K mutation broadens APs and decreases the neuronal firing rate when transfected into DRG neurons.

### Nav1.8 M650K shifts voltage dependence of steady-state fast inactivation to more hyperpolarized potentials

**Fig 4:**
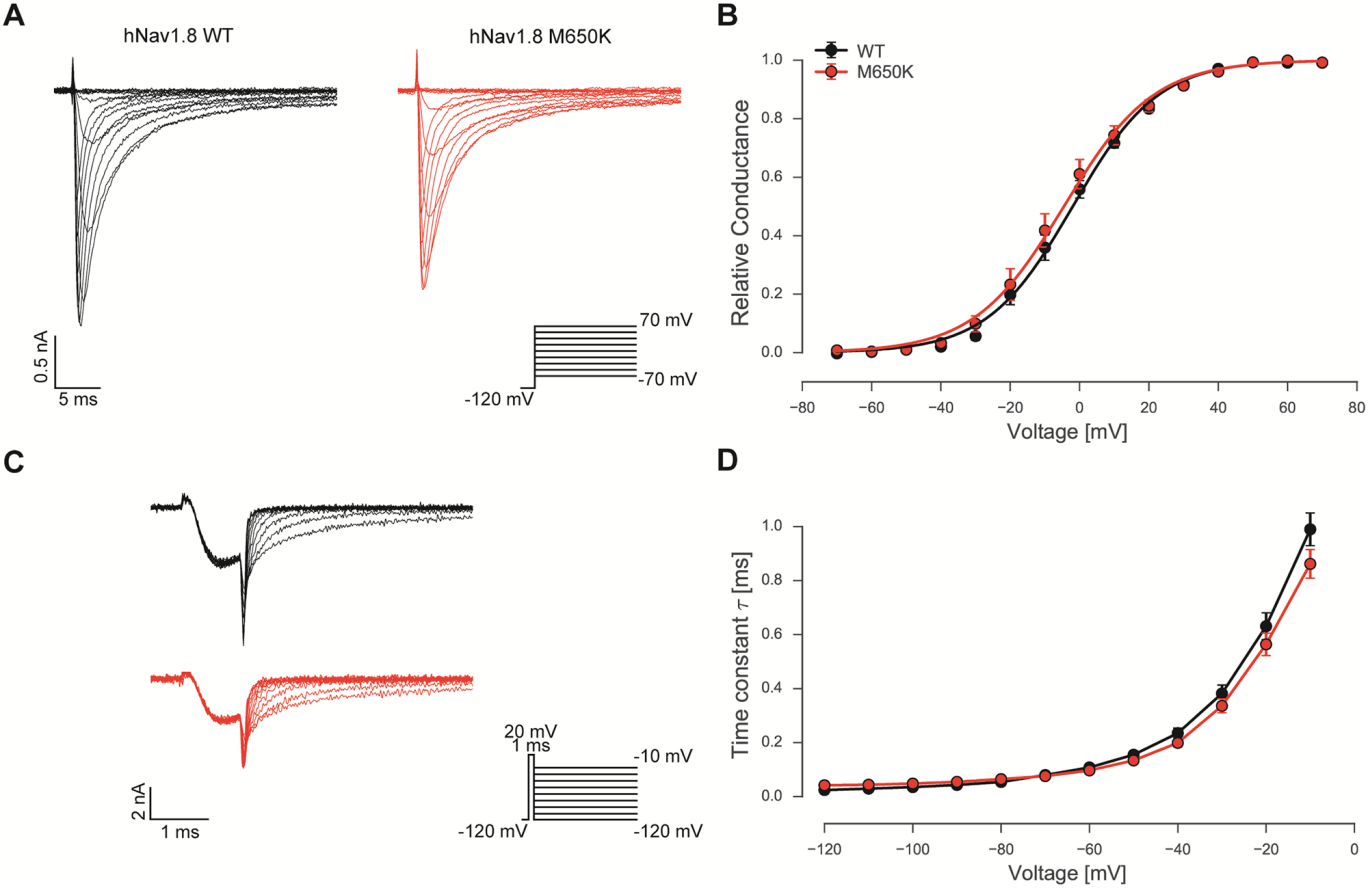
Voltage-dependence of activation and deactivation of Nav1.8 is unaltered by the M650K mutation. A: Pulse protocol and representative voltage-clamp recordings of hNav1.8 WT and hNav1.8 M650K expressed heterologously in ND7/23 cells. Traces were digitally low pass filtered for display.B: Conductance-voltage relations for WT (black circles, n = 10) and the Nav1.8 M650K mutation (red circles, n = 12) are shown. Solid lines represent Boltzmann-fits to the mean data points. C: Pulse protocol for deactivation measurements and representative recordings of hNav1.8 WT and hNav1.8 M650K mutant expressed heterologously in ND7/23 cells. D: Deactivation time constants as derived from single exponential fits of current decay during repolarization for WT (black circles, n = 13) and M650K (red circles, n = 15) at various voltages.

To assess Nav characteristics that may lead to the observed broadened AP, we transiently transfected Nav1.8 and its M650K mutation into ND7/23 cells. By adding 1 μM TTX to the bath solution we blocked all endogenous Nav currents and thus recorded Nav1.8 in isolation using voltage-clamp (Fig 4). Expression of Nav1.8 was not affected by the mutation (current density at +10 mV was -162 ± 15 pA/pF for WT (n = 10) and -171 ± 11 pA/pF for M650K (n = 12), shown in S1 Fig).

Nav1.8 activated and inactivated within 20 ms (Fig 4A) and introduction of the M650K mutation did not alter voltage-dependence of activation (Fig 4B). The voltage of half maximal activation derived from Boltzmann fits to the conduction-voltage curves revealed a V_half_ = -2.1 mV ± 2.68 mV (WT) and -4.7 mV ± 1.53 mV (M650K) and a slope of 11.75 mV and 11.46 mV for WT and M650K, respectively. Broader APs like those recorded in M650K transfected DRG neurons may be due to slowed deactivation kinetics. We assessed deactivation using a brief depolarization of 1 ms to +20 mV in order to fully activate all expressed channels, followed by hyperpolarizing pulses to potentials between -120 mV and 0 mV (Fig 4C). Time constants derived from a single exponential fit to the deactivating currents were not significantly different between WT and M650K mutation at all potentials tested (Fig 4D). The remaining mean persistent current measured between 80 and 100 ms during the test pulse were not affected by the mutation (S2 Fig).

**Fig 5:**
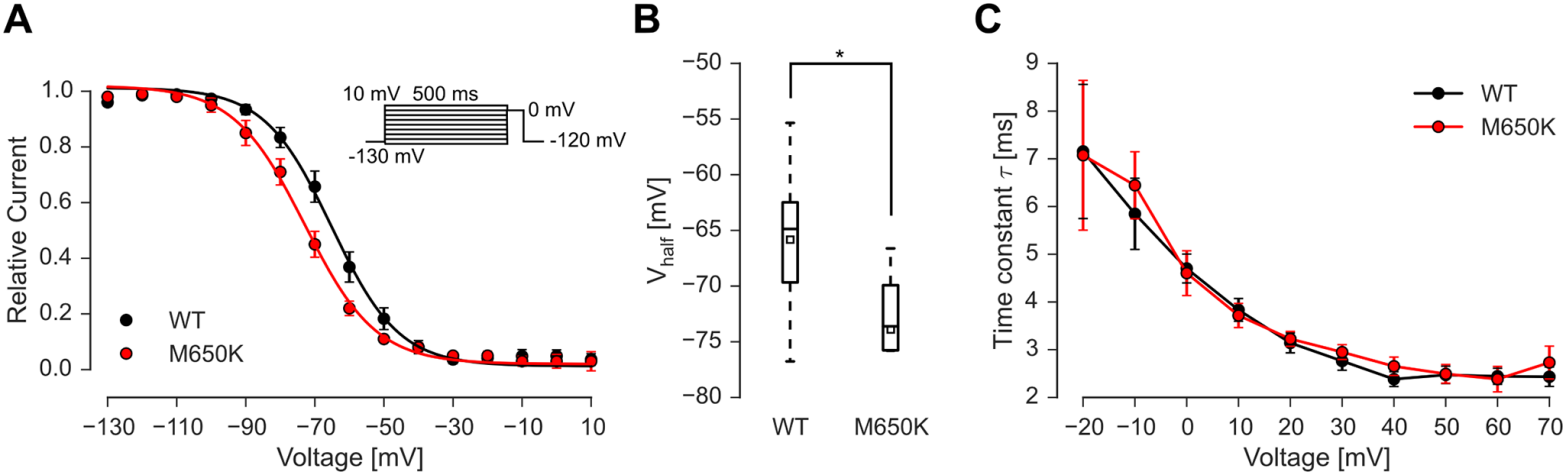
The M650K mutation shifts steady-state fast inactivation to more hyperpolarized potentials. A: Voltage dependence of steady-state fast inactivation as determined with the indicated pulse protocol. Black circles indicate WT (n = 7–8) and red circles the M650K mutation (n = 7–8). B: Distribution of the V_half_ determined by Boltzmann fits to each cell. Mean is indicated as an open square, the median is depicted as a line inside the box, the boxes indicate 50% confidence interval and whiskers indicate 95% confidence interval. C: Time constants of single exponential fits of currents evoked with the pulse protocol shown in Fig 4A did not differ between WT and M650K mutation at any investigated voltage.

In contrast, the voltage-dependence of steady-state fast inactivation was significantly shifted to more hyperpolarized potentials (Fig 5A). Mean V_half_ revealed by Boltzmann-fits for WT and M650K mutant were -66.0 mV ± 6.5 mV and -73.8 mV ± 2.2 mV (slope: -9.03 mV and -9.66 mV), respectively (Fig 5B). Inactivation decay kinetics, as determined by a single exponential fit to current decay using depolarizing pulses (as shown in Fig 4A) did not show any differences between WT and the M650K mutation (Fig 5C).

Recovery from fast inactivation as determined by an increasing inter-pulse interval at -120 mV and at -70 mV for periods between 1 ms and 512 ms did not reveal any significant changes induced by the M650K mutation (n = 13 for WT and n = 11 for M650K, S3 and S4 Figs). Similarly, slow inactivation as measured following a 30s pre-pulse revealed no shift of its voltage-dependence nor its slope (n = 6 for WT and n = 3 for M650K, S5 Fig). Thus, our electrophysiological results show a broadened AP and a shift of steady-state fast inactivation to more hyperpolarized potentials.

## Discussion

Here we present a patient diagnosed clinically with EM based on typical symptoms, such as burning pain that was relieved by cooling. Sequencing of Nav1.7, the main candidate gene for inherited erythromelalgia and several other candidate genes revealed no mutation [8]. Related diseases, medications or toxic influences causing secondary EM were also absent. However, the subtype Nav1.8 contained the missense mutation p.M650K. Current clamp recordings of DRGs transiently transfected with WT and M650K hNav1.8 displayed a broader AP and reduced firing rate upon current injection. In agreement with this, voltage-clamp recordings revealed a hyperpolarizing shift of voltage-dependence of steady-state fast inactivation induced by the M650K mutation. Microneurographic recordings showed not only spontaneous activity and mechanical sensitization of some CMi fibers, but also CM-nociceptors responding only weakly to mechanical stimuli and CMi fibers with large ADS, which may be indicative of a reduced ability of the fibers to fire at higher frequencies over longer time periods.

Mutations of Nav1.8 were reported previously to be linked to SFN patients [33,34]. These results suggested a causative link between the Nav1.8 mutations and the disease based on the observed hyperexcitability of cells and fibers expressing the mutant channel. The authors described four additional mutations of Nav1.8 in SFN patients, for which no electrophysiological analysis was performed. A basic investigation of A-fiber nerve conduction velocity in these patients did not show any abnormalities; ADS of C-fibers was, however, not determined [33]. Choi et al. (2010) described no change in excitability induced by a mutation in Nav1.8 in a patient with erythromelalgia who had additionally a gain-of-function mutation in Nav1.7 [24]. Another study showed that the G1662S mutation in Nav1.8 in a patient with SFN is responsible for a 7 mV shift of the steady-state fast inactivation to more depolarized potentials causing hyperexcitability [35]. We observed a shift of the same magnitude, however, in the opposite direction, suggesting a reduced excitability of C-fibers.

In microneurography experiments we observed CM fibers, which resemble polymodal nociceptors in rodents, with an apparent low response to mechanic stimuli suggesting a hypoexcitability of the fiber, which does not allow for high frequency bursting. Nav1.8 is thought to support bursting or higher frequency firing [20] and Nav1.8-expressing neurons are required for inflammatory, cold, and mechanical pain [15].

We observed a lower firing rate of DRG neurons transfected with the Nav1.8 M650K mutation. In this respect, patch clamp data and microneurography findings are compatible. Additionally, a hyperpolarizing shift of steady-state fast inactivation of the M650K was observed using transfected neuroblastoma cells. Resting membrane potential of neurites of sensory neurons was measured to be around -60 mV [36]. At this potential, 77% of the M650K mutated Nav1.8 is fast-inactivated, compared to only 67% of WT channels (Fig 5). Thus, the M650K mutation is reducing the number of available channels. This may underlie the lower AP frequency observed in current clamp (Fig 3). It may also result in the observed discharge patterns in microneurography experiments, such as the weak response to mechanical stimuli and the relatively low spontaneous activity. In control patients with EM lacking a mutation in Nav1.7 and 1.8, a higher incidence of spontaneously active fibers was found compared to the patient with the M650K mutation (42% versus 10%). Both patient groups were examined in a relatively painless period and not during a full pain attack. This is paralleled by the relatively moderate pain-phenotype of the M650K patient. The mean pain intensity during attacks in this patient was felt as 5 on a scale from 0 to 10 on a numeric rating scale while other EM patients describe their pain attacks between 6.5 and 10 [5].

Interestingly the patient had normal thermal thresholds in QST in the relatively pain free interval. This could be due to the fact that there is a difference in the mechanisms determining sensory thresholds of natural stimuli and the mechanisms leading to ongoing pain [5]. The potentially protecting nature of the mutation in Nav1.8 leading to hypoexcitability might likewise play a bigger role in regulating ongoing activity than in determining thresholds. Thresholds may depend more on Nav1.7 function and are the result of an integrative function of fiber number, fiber function and subsequent activation and silencing of fibers i.e silencing of cold fibers by warmth being responsible for the feeling "warm".

The patient did not fulfil the objective diagnostic criteria for polyneuropathy and since thermotesting was normal, we were also unable to find support for a diagnosis of small fiber neuropathy except for the fact that the patient suffered from pain. A puzzling detail is the reduced sensitivity to light touch on the feet. However, it was shown that Nav1.8 is also expressed in large fibers [37]. Thus the reduced firing frequencies observed in current-clamp recordings could also occur in large fibers and account for the reduced sensitivity to light touch. In addition, the patient may develop peripheral nerve fiber degeneration over time, which is currently not pronounced enough to be detected in electrophysiological tests. Nevertheless, it may appear as reduced touch sensitivity due to potentially reduced firing function of the fibers expressing the Nav1.8 mutation. Broader APs lead to increased sodium ion influx which may result in a reverse activity of the Na^+^/Ca^2+^ exchanger and thus increase intracellular calcium ion concentration [38]. This may support fiber degeneration.

When compared to controls or to patients that suffer from erythromelalgia without a mutation in Navs, ADS of the M650K patient seemed to be enhanced in CMi fibers. However, ADS in this specific control group of EM patients may be reduced in comparison to healthy age matched controls ([5]; Fig 2). The finding of pronounced ADS and especially the finding of two CMi fibers with a progressively increasing ADS during 2 Hz stimulation might be directly linked to the mutation. This progressive increase of ADS is rarely observed in other neuropathy patients or healthy subjects. ADS is thought to be based on slow inactivation of Navs and accumulation of sodium ions intracellularly and thus linked to the excitability of the axon [11,39]. Thus, ADS acts as some kind of negative feed-back mechanism that limits longer lasting or high frequency discharge. Pronounced ADS might even lead to a complete block of AP conduction (Col et al., 2012). Broader APs in the M650K mutation would increase sodium ion influx per AP which supports sodium ion accumulation in the neurite. This shifts the Nernst potential for sodium ions to less positive potentials and thus can be linked to increased ADS [40]. CMi fibers are thought to express more Nav1.8 than CM fibers [11,14]. Thus, the mutation in Nav1.8 should have pronounced effects on CMi fibers, and indeed the increased ADS in this patient was significant only in CMi fibers, but not in CM nociceptors.

The results of our patch clamp analysis might explain reduced nociceptor excitability in pain-free intervals, but are seemingly at odds with the occurrence of pain attacks. As sensory DRG neurons express several Nav subtypes [41], Nav1.8 could play a partial role in evoking spontaneous pain and may serve as a tuning channel as proposed by Petersson et al. [40]. Hyperpolarization of the RMP induced by warming may help Navs to recover and support AP firing. Enhanced inactivation of Nav1.8 additionally supports hyperpolarization of the cell membrane after an AP. Thus, mechanical sensitization and spontaneous activity of C-fibers of the M650K patient could be indirectly achieved, as enhanced fast inactivation of the M650K mutation of Nav1.8 may support recovery of TTXs channels. Thus, more TTXs Navs would be available and fibers with the M650K mutation would be more sensitive to subthreshold stimuli.

We cannot rule out that the Nav1.8/M650K mutation has a protective effect and that the patient would suffer from her IEM even more, if she did not have the described mutation. Nav alpha subunits may also interact with each other and it was shown that Nav1.8 is in direct physical contact with Nav1.5, thereby enhancing sodium current density [42]). A mutation in Nav1.8 associated with the cardiac arrhythmia Brugada syndrome seems to alter this interaction, and thus reduces current density when co-expressed with Nav1.5. It is possible that a similar mechanism is at play in Nav1.8/M650K mutation as well, and that the surface expression of other sodium channel subtypes in nociceptive neurons is altered, and thus excitability is affected. To date no such interaction with other Nav channels than Nav1.5 was shown and studies are needed to investigate this possibility. However, spontaneous burning pain during the attacks does not entirely fit with the reduced firing rates observed in current-clamp experiments, the increased ADS and reduced response of the nociceptors to mechanical and heat stimuli. In addition to these peripheral changes there may also be an effect on the synaptic transmission process in the dorsal horn. A broader AP allows for more sodium and calcium ion influx per AP and thus could lead to facilitated neurotransmitter release at the first synapse in the dorsal horn [43–45]. This would also result in substantial transmission of low frequency input i.e. from spontaneously active fibers or mechanically sensitized nociceptors and thus possibly to pain despite the ability of the nociceptor to fire only low frequencies.

Taken together, we found correlations between microneurography findings and patch clamp results from a patient with primary erythromelalgia carrying a Nav1.8 mutation. A broader AP could contribute to increased ADS via increased sodium influx and thus more pronounced negative feed-back of nociceptor activity under resting conditions. This was observed mainly in CMi nociceptors, which are thought to express more Nav1.8 than other fibers. However, a broader AP could also lead to more reliable synaptic transmission in the dorsal horn, perhaps contributing to the pain during the attack, when the transmission of low frequency input via spontaneously active primary afferents is amplified. The changed Nav1.8 kinetics could underlie the lower firing rates observed during voltage clamp and parallel the reduced mechanical responses in nociceptors during microneurography. In summary, the changes in neuronal function in this patient, as determined by patch clamp analyses and single nerve fiber recordings correlate well with clinical findings.

## Supporting Information

S1 Fig The current density is not different between WT (N = 10) and M650K mutant (N = 12). Here, we define current density as the maximum inward current divided by the measured cell capacitance. Current density at +10 mV is -162 ± 15 pA/pF for WT (n = 10) and -171 ± 11 pA/pF for M650K (n = 12).
(TIF)

S2 Fig The persistent current is not different between WT and M650K mutant (n = 7 and n = 11, respectively). Persistent current decreases towards the Na^+^ reversal potential. Here, we measured the persistent current as the mean inward current 80 to 100 ms after the step depolarization (compare inset).
(TIF)

S3 Fig Recovery from fast inactivation is not altered between WT (n = 13) and MK (n = 11) with a holding potential of -120 mV. (TIF)

S4 Fig Recovery from fast inactivation is not altered between WT (n = 12–13) and MK (n = 11–12) with a holding potential of -70 mV. (TIF)

S5 Fig Slow inactivation is not altered between WT (n = 6) and M650K (n = 3) mutant. Slow inactivation was measured as the ratio of pre-pulse and test pulse (depolarization to +40 mV) after a 30 s pulse forcing the channels to undergo slow inactivation.
(TIF)

S1 File Excel-Sheet containing an overview with Excel generated figures close to the ones presented in this manuscript, and all recordings leading to the figures. (XLSX)

S2 File Table containing the single data presented in the tables in the manuscript. (PDF)
