# Supplementary figures and images for: SCN10A Mutation in a Patient with Erythromelalgia Enhances C-Fiber Activity Dependent Slowing
Source: PLoS One. 2016 Sep 6;11(9):e0161789. doi: 10.1371/journal.pone.0161789 (PMC5012686; doi:10.1371/journal.pone.0161789)

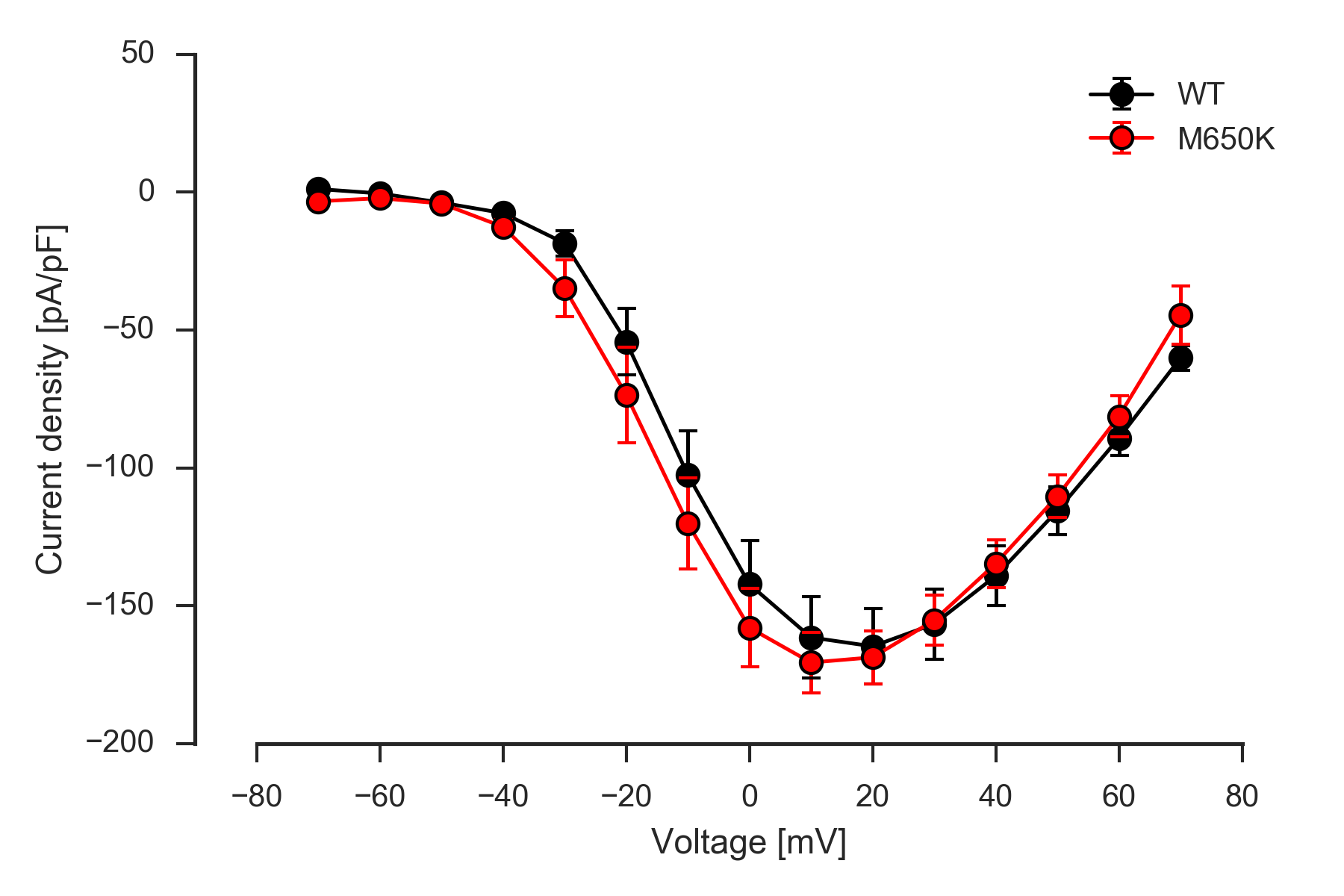

Supplement: S1 Fig — Here, we define current density as the maximum inward current divided by the measured cell capacitance. Current density at +10 mV is -162 ± 15 pA/pF for WT (n = 10) and -171 ± 11 pA/pF for M650K (n = 12). (TIF) [file pone.0161789.s001.tif]

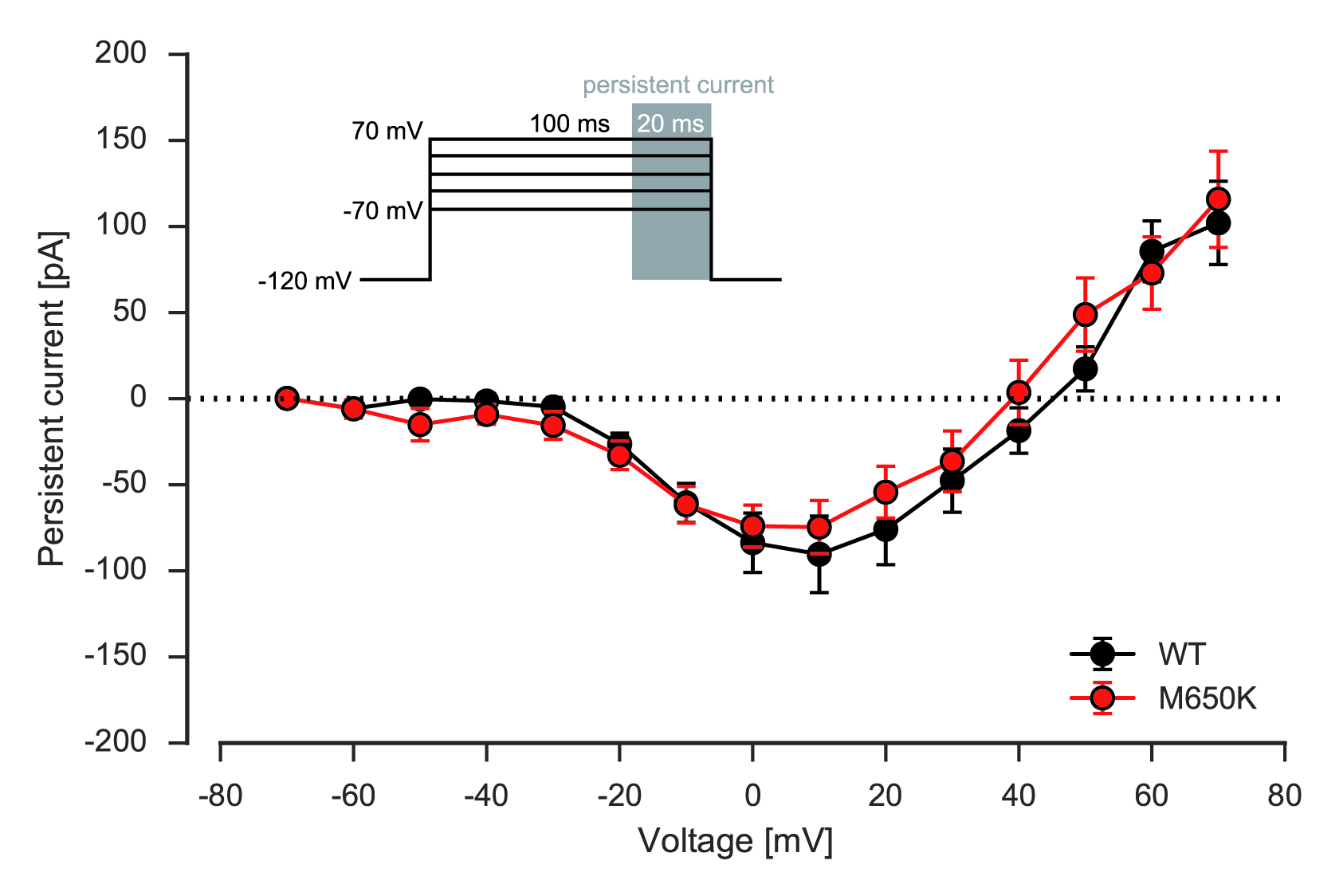

Supplement: S2 Fig — Persistent current decreases towards the Na+ reversal potential. Here, we measured the persistent current as the mean inward current 80 to 100 ms after the step depolarization (compare inset). (TIF) [file pone.0161789.s002.tif]

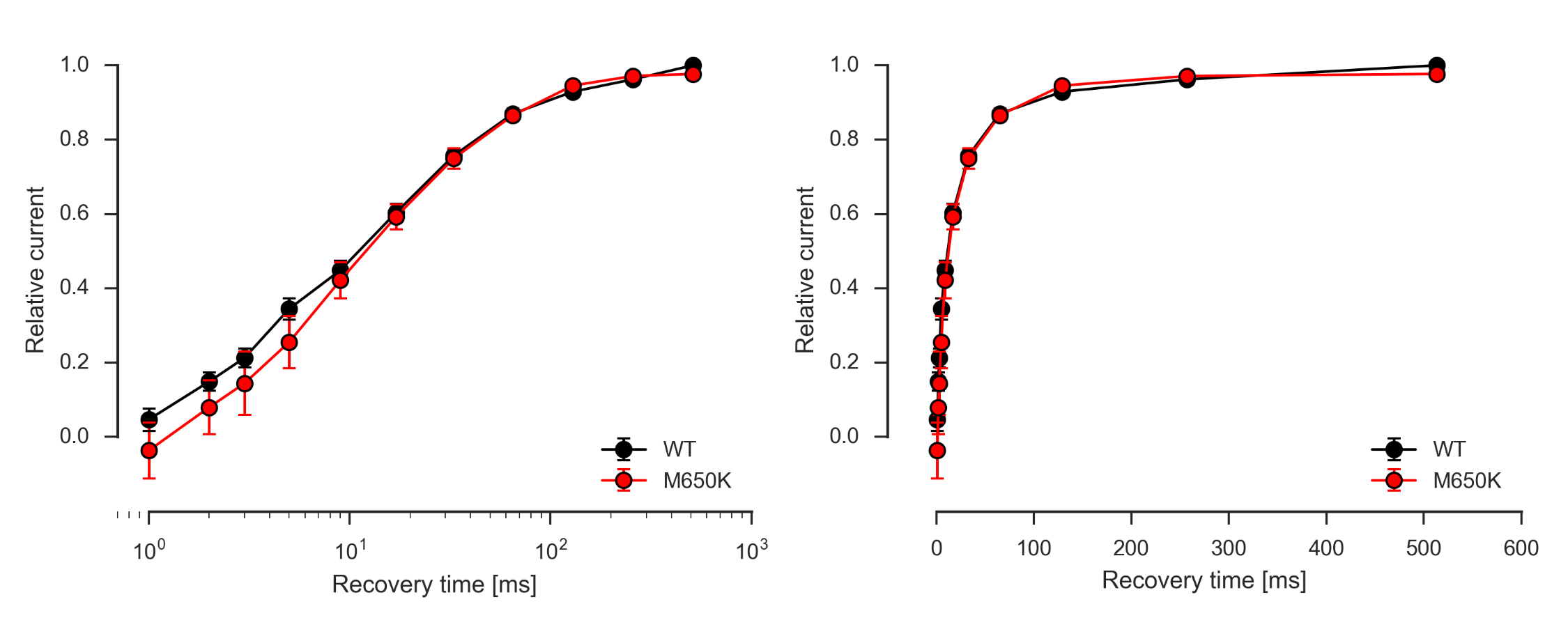

Supplement: S3 Fig — (TIF) [file pone.0161789.s003.tif]

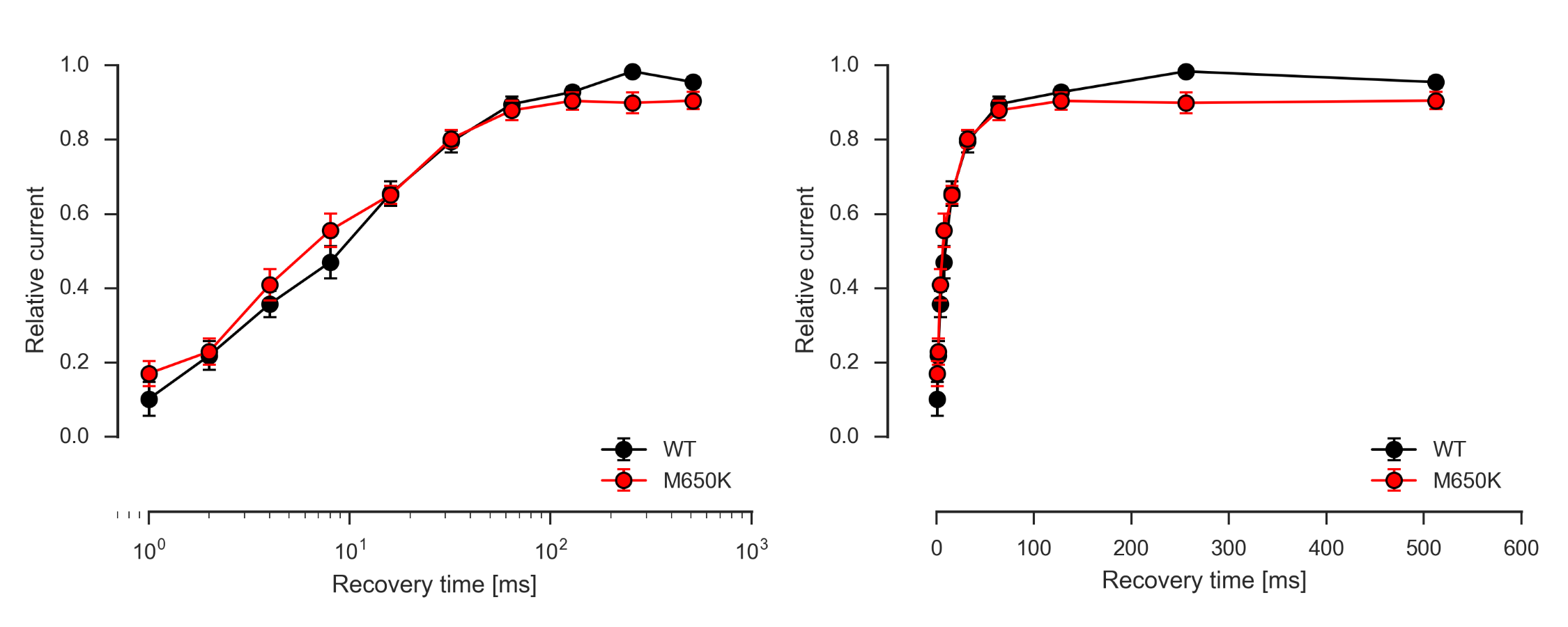

Supplement: S4 Fig — (TIF) [file pone.0161789.s004.tif]

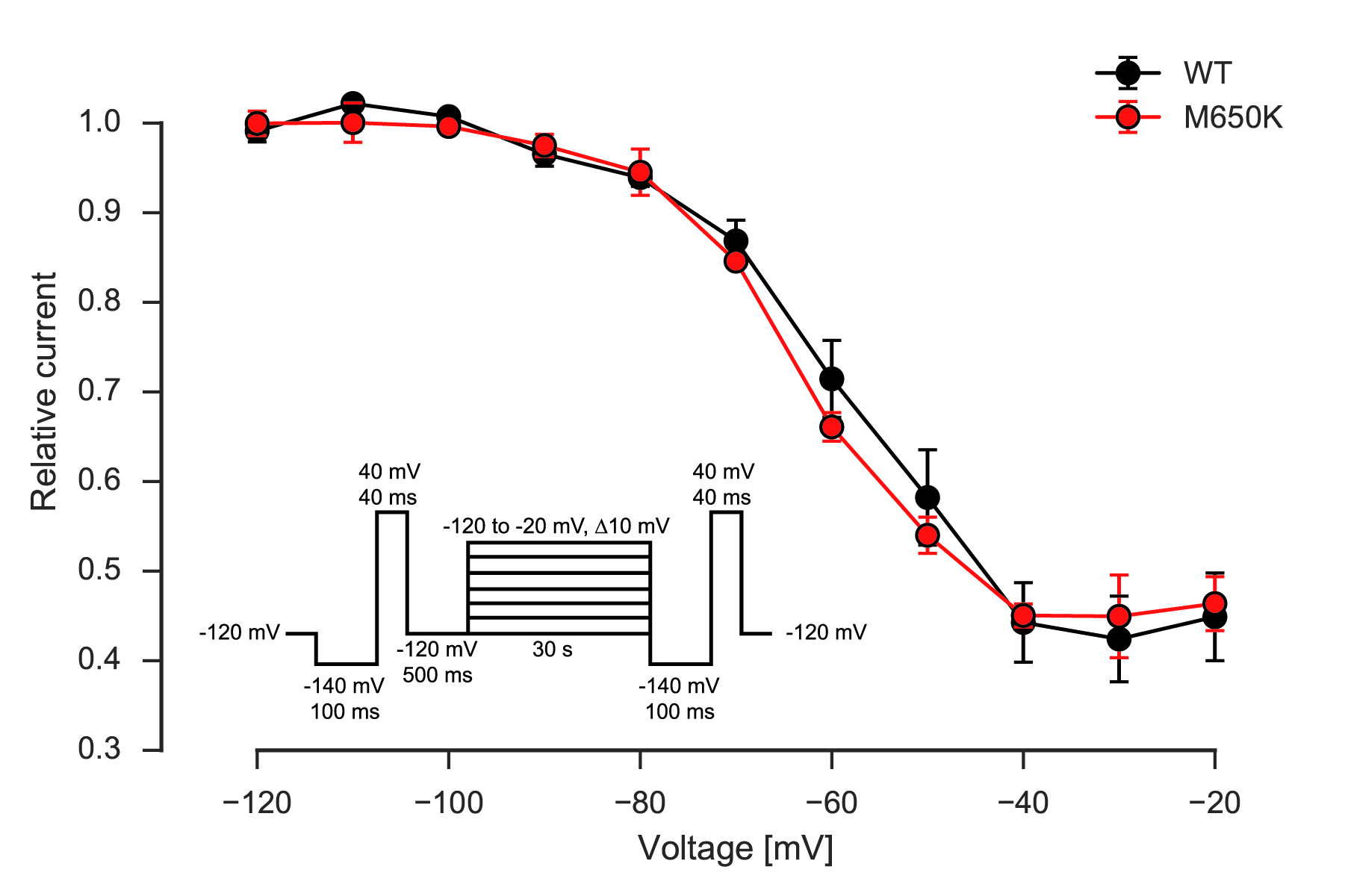

Supplement: S5 Fig — Slow inactivation was measured as the ratio of pre-pulse and test pulse (depolarization to +40 mV) after a 30 s pulse forcing the channels to undergo slow inactivation. (TIF) [file pone.0161789.s005.tif]
