# Supplementary material for: SCN10A Mutation in a Patient with Erythromelalgia Enhances C-Fiber Activity Dependent Slowing
Source: PLoS One. 2016 Sep 6;11(9):e0161789. doi: 10.1371/journal.pone.0161789 (PMC5012686; doi:10.1371/journal.pone.0161789)
Supplement: S2 File — (PDF) [file pone.0161789.s007.pdf]

CM_healthy_ADS1/8	CM_healthy_ADS tot	CM_healthy_cv	CMi_healthy_ADS1/8
0,430686406	1,830417227	1,049798116	1,184068891
-0,854160076	1,486871243	0,711800063	0,92879257
0,444983819	3,05420712	1,011326861	0,892857143
0,702151755	2,718006795	0,928652322	0,420248329
0,2852754	2,611367127	0,899714725	2,55839822
0,281690141	1,920614597	1,04993598	2,220812183
0,479846449	2,471209213	0,983685221	3,069520491
0,327408793	2,502338634	0,958840037	2,027345592
0,325884544	2,420856611	0,954376164	2,678720911
0,175262894	2,278417626	0,685274946	2,069068175
0,17699115	2,101769912	1,02653981	4,458354624
0,300816502	2,965191233	0,907079646	1,4111734
0,181598063	2,481840194	0,862976216	1,174277726
0,594059406	2,347949081	1,02905569	2,018153727
-0,424005219	1,272015656	0,961810467	1,616272677
0,386100386	3,474903475	1,108936725	2,396284176
0,472440945	4,094488189	0,965250965	0,821052632
-0,106202209	1,677994902	0,787401575	2,033940604
1,010710514	4,314376226	0,892098556	1,518942886
0,141442716	1,697312588	0,663750189	3,205643914
0	3,43217371	0,961810467	2,100505185
-0,03312946	2,155963303	0,895915679	2,131519274
0,479413424	1,240834743	0,793836096	0,680619364
0,089928058	0,899280576	1,044852192	1,94105979
-0,158290463	1,028888009	1,156232375	0,399828645
0,088731145	1,197870453	0,92176259	1,784243755
0,019278967	1,657991132	0,811238623	1,780334155
0,175394638	1,854171887	0,998225377	1,179941003
0,576296668	2,405412177	0,963948332	2,095792677
0,205620288	1,576422207	0,914557755	1,659751037
0,220507166	1,653803749	1,277875219	10,52344602
0,325450206	2,64699501	1,005254741	1,364113326
0,252873563	2,781609195	0,970231533	1,839348079
0,123670542	3,141231759	0,954653938	1,59512761
0,241254524	3,015681544	0,942528736	1,944110623
0,117508813	1,292596945	1,014098442	1,776530243
0,182982617	2,996340348	0,989143546	
0,428410372	3,404735062		
0,463678516	3,576948554		
0,26172301	2,726281352		
0,371287129	3,589108911		
0,220804711	2,870461237		
0,221595488	3,40451249		
0,195121951	1,697560976		
0,422960725	3,726082578		
1,219268439	3,63781731		
0,416204218	2,413984462		
0,24219591	4,117330463		
0,061211998	1,387471945		

0,074589756	1,616111387
0,799175045	2,294405775
0,35035035	1,601601602
0,203183204	2,031832035
0,337623971	2,679890272
0,354706685	2,07366985
0,259924386	1,512287335
0,394650296	2,872177154
0,282558438	3,236578474
0,25083612	3,210702341
0,187090739	2,432179607
0,333044983	3,871107266
0,091340884	1,680672269
0,18496721	1,96737851
0,264284758	2,8703332
0,381367475	2,01579951
0,021748586	1,979121357
-0,044540378	1,202590194
-0,010607266	2,916998144
0,224071703	2,272727273
0,247770069	1,833498513
0,716845878	4,356217259
0,19846192	2,282312081
0,30261348	2,640990371
0,40660737	3,608640407

CMi_healthy_ADS tot	CMi_healthy_cv	CM_EM_ADS1/8	CM_EM_ADS tot
6,630785791	0,8934338	SLOW1/8)%	SLOWING_TOTAL %
6,08875129	0,856553148	0,672	2,426
6,392857143	0,741071429	0,032	0,996
5,692454632	0,343839542	0,227	1,668
10,96774194	0,500556174	-0,047	1,637
9,877326565	0,867174281	-0,099	1,125
10,98390576	0,829600133	0,114	0,696
12,49410655	0,730787364	0,300	1,178
10,66465763	0,686422233	0,506	2,003
9,705269425	0,64007145	0,276	1,949
11,52273889	0,549310169	0,302	2,370
6,611250725	0,908563696	-0,110	0,397
6,467847158	0,894687791	-0,022	0,954
9,762456547	0,463499421	-0,041	1,019
10,64321056	0,483782298	0,050	2,413
10,70410641	0,46447799	-0,021	1,023
8	0,715789474	0,213	1,804
7,44571999	0,66134265	0,426	1,552
6,598182537	0,89995171	0,147	1,823
12,04282443	0,377498608	0,181	3,621
7,41823983	1,010369583		
10,38548753	0,861678005		
6,091543304	0,757189042		
6,687446869	0,935109096		
7,882336142	0,614022562		
8,18007137	1,070546253		
8,189537113	1,068200493		
8,259587021	1,209439528		
8,644161158	0,537719022		
8,352877503	0,46003969		
13,22246456	1,022355507		
6,67366212	0,786988458		
8,009313155	1,187427241		
7,337587007	1,189095128		
8,987328293	0,753000593		
8,442593263	0,742484607		

CMi_EM_ADS1/8	CMi_EM_ADS tot	CM_1.8Mut_ADS1/8	CM_1.8 Mut_ADS tot
1,521	7,108	0,23988006	1,484257871
1,067	6,902	0,250065807	1,3556199
2,337	8,844	0,280269058	1,644245142
0,888	6,420	0,251968504	2,803149606
1,233	6,460	0,135611608	1,017087063
1,375	6,148	0,376789751	1,557397639
1,608	7,073	0,176470588	2,4
1,061	5,465	-0,108264165	0,613496933
		0,220264317	3,132648067

CMi_1.8Mut_ADS1/8	CMi_1.8Mut_ADS tot
2,044161604	12,46745733
1,955525757	8,749580959
1,922635006	9,314438912
1,260504202	6,892230576
1,005961252	6,287257824
0,761394102	4,857908847
1,257800312	6,39625585
1,280866235	5,889343721
